# Foreign Observations

**Published:** 1902-01

**Authors:** Frederick Bradley

**Affiliations:** Newport R. I.


### "FOREIGN OBSERVATIONS."[1]

BY FREDERICK BRADLEY, D.M.D., NEWPORT, R. I.

Mr. President and Gentlemen,—I came near being disappointed in being able to present anything to you to-day. I was especially fearful of this when I was on the good steamship Kaiser Wilhelm and the custom officials came around. It was a question

---

[1] Read before the Harvard Dental Alumni Association, June 24, 1901.

whether I should be able to bring anything except myself. In fact, so closely are the passengers questioned, that it is related of one lady who was returning with a small baby of some two months old, it was a question as to whether she would be allowed to land without paying duty, as the officer was not sure but that it was an importation. It is also a fact that my daughter was questioned when she appeared on the wharf with some little knick-knacks which she had to prove she had bought in New York.

As a result of my observations in Europe and England, I have come to three or four conclusions:

1. The American dentist in Europe is a most agreeable person to meet, as I have met him; and I may say, also, the native dentist of each country was courteous and desirous of making my stay in his city or town agreeable as well as profitable and interesting. The gentlemen whom I met I made a practice of asking them the condition of the profession in the various places, also as to the treatment of the poor, the care of the teeth of the poor children, as well as to the education of the student, and will refer to it again later.

All the Americans, with one or two exceptions, whom I had the pleasure of meeting, seemed to have developed a large and lucrative practice. Their rooms were large, well, if not luxuriously, furnished, especially on the continent, and every indication of success was plainly written; yet with it all they were distinctly American in sentiment and feeling. Every one looked forward to that future, and thought of their positions as an end to be accomplished, as a means towards the end. They all probably look forward to educating their children in America. They want them to be American citizens and not citizens of France or Germany. They look to America as their homes.

2. The laity in every country cordially conceded the palm of superiority to the American dentist; everybody, high or low, who has ever sought the services of a dentist, intimating his purpose to put himself only in their hands. By that I do not mean to say that they go regularly as they should, for they have not yet been educated up to that position where they will go regularly to the dentist,—they now go only when driven there. A great many patients after a recent treatment, if the tooth feels comfortable, will not go until it has given them considerable trouble. But they do not like to pay for broken engagements. Of course there are dentists—natives of every country—who are successful; many of them have, however, been educated in the States professionally, and no doubt they make the most of that fact, as their patients seem to feel that it is a reason sufficient to rest in. I do not doubt that the large majority of the cultured, intelligent, and wealthy people of every country consider themselves as patients in the care of an American dentist or one educated in America.

We find, however, that there is an inclination on the part of some of the foreign dentists to treat us as we treat foreign labor. They are somewhat afraid of them, for fear that they may perhaps take away their trade or business; and the native dentist seems to feel in that way in regard to an American dentist settling there. Sometimes it is very uncomfortable to feel that you are regarded in this way, but the moment it was found out that I was there merely on a visit, in Paris especially, I was introduced to a number of dentists and treated with the utmost courtesy. They could not do enough in the way of introducing me when they knew I was not going to settle there. For instance, in Naples and in Milan, the native dentist seemed to be a little bit shy about making the acquaintance of the American dentist, but all this disappeared when I said I should never think of coming there to enter into practice. Then they expressed themselves as being highly pleased to meet me; but I think they showed a little envy of the success of the American dentist. This brings me to my next conclusion.

3. That the dentist who is educated in Europe—what might be termed the native European—is to a certain extent and in a certain way jealous of or envious of his American *confrère*. This feeling is difficult to define or to explain, but I am confident many Americans have realized the presence of this feeling, and will agree with my statement. This brings me to my next conclusion.

4. That the trend of recent legislation in Europe is to add to the difficulties in the way of the American dentist securing a license to practise, or a position in the profession. I was unable to find any place where a diploma granted by a dental school in the States would be accepted by the authorities and a license to practise granted.

Of course, we in this country have to go before the State Board; but it seems to me that a diploma which is granted by a reputable school ought to indicate at least that they are in a position to take the examination, but on the European continent and in Great

Britain there seems to be no such an intention. This is far from the facts of the case. They require seven years' study, and depend on the time which is given to it. One must be in the hospital so many years, and in this and in that so many years. It is very difficult to get this license, and the laws seem to make it more difficult, in fact, to make it as difficult as possible. Besides, their examinations are not upon dental subjects, but upon surgery and medicine, and really upon almost everything but dentistry. Of course, a diploma does not secure a license in this country, but it is supposed that the necessary examination will cover only the studies taken up in the average dental school; in Europe, on the continent, especially marked attention is given to strictly medical and surgical questions, matters which of themselves do not fit a man any better for his work. Of course such broad culture is desirable as a liberal education is desirable; and if such legislation had been enacted solely for the purpose of raising the professional standard, we should be the last to complain, assistants or otherwise.

I will speak further of what I would suggest if you are thinking of going abroad. Within a short period it has been the custom for a reputable dentist who had a license from the country to take as an assistant an American dentist without any examination whatever, and it would in that way not be necessary for him to have a license. Now, every assistant must pass an examination before he can go with any one else to do anything except some laboratory work.

Now, a few words as to the educational institutions which I have visited. There are a number of schools devoted to dentistry, some of which I visited. In Berlin, Dr. Miller is an enthusiastic worker as the head of the school there, making the most of their facilities, operative dentistry being carried on in a number of small rooms, with chairs crowded together, imperfect light, and I should judge rather limited oversight or instruction, yet the students are much in earnest, and no doubt they will accomplish something. Dr. Miller is planning for and expecting a new building in the near future, and a part of his work in visiting this country is to examine buildings and facilities which may be of service in Berlin. Dr. Miller was particularly kind and courteous to me when I was in Berlin.

Dr. Miller is about to visit the States this summer. He will be here next month probably, visiting us in Boston, and he is looking forward to receiving many suggestions from us in the putting up of the new building in Berlin. I told him what we were doing, and

I hope that some of us will have the pleasure of hearing him. He is a very busy man, and I have come to the conclusion that the very busy man is the man who accomplishes the most in this world. I found that Dr. Miller was not so busy but that he could give me a few minutes, and I found him remarkably solicitous to do what he could to make my short visit in Berlin as interesting as possible.

In Paris I visited a school in charge of Dr. Godon, the Dean. They had a large operating-room lighted from the ceiling but not from the walls, and this gave every chair in the room an equal opportunity as regards the light; each student had an equal chance of the light, and they were working very earnestly there. In this school I saw an extensive collection of models and appliances showing loss of tissue from various diseases or other causes, and the means taken to restore the parts lost, and some of the appliances used were very ingeniously arranged. Everything seems to have been done in a hurry, and they are not careful enough in making examinations. In London I saw a student, without any apparent oversight, very busy making a large filling for an inferior cuspid tooth, and he had gotten pretty far along. I noticed that there was very little space between the teeth, and I asked him how he would treat the mesial surface of the first bicuspid, which had a small cavity in it. He did not seem to realize the necessity of filling that, and he was covering it up. He called the instructor's attention to it, and he decided that on the whole it was better to fill it.

In London I visited the National Dental School, but was unable to see the Dean. The young gentleman in charge assured me there was nothing in London superior to this school. I found afterwards, too late to visit it, the Leicester Square School had just put up a fine new building; in fact, it is not yet finished. I did not get an opportunity to visit this school, because I left Europe, on my return, the next morning after I had heard of it, and I was greatly disappointed in not being able to do so. In all the schools I visited, marked attention was given to anæsthetics and extraction, and there are always free patients. They average eighty-five per day in extracting, and I do not know how many in filling teeth. One instructor boasted of the large number of extractions daily. All the schools find patients for the free clinics, as they are called, just as we do. In many large cities there is no dental school or infirmary, but the poor have the services of a visiting dentist or his assistant at the surgical department of the city or town hospital. For instance, in many places I was unable to find any institution for infirmary work, and yet the poor can visit the regular hospital, and there will be some visiting dentist to perform extractions, but do nothing in the way of filling teeth. In such places as Naples and other large cities I found that some of the laboratory assistants in the American dental offices had started a little dispensary for the purpose of practising upon the poor, and they had worked up a little practice among these people by this cleaning and filling for practice.

My observation would lead me to think that the teeth of the poorer classes in Italy were above the average in quality, and in Great Britain, especially in the manufacturing districts, the same classes seemed to have utterly neglected their teeth so far as regards cleaning or preserving them is concerned. As I have just said, the poor people of Italy have as good teeth as any people I saw, and among the manufacturing people of England have the very worst, and they are the most careless of any people I saw.

It is generally conceded that the more refined classes are becoming interested in the salvation of their own teeth; but there yet remains much to be done to fully convince even those of the necessity of regular and frequent attention at the hands of the dentist. There is an abundance of work to be done; there is great need of competent men, but there is no place for any one who cannot pass the required examination, and such men as cannot comply with this requirement have a very hard and disagreeable time and bitter experience.

What shall be done? I would suggest to the Executive Committee that they find out the exact requirements in each country, what is required for a man to practise, and particularly to get a copy of the law. I tried to get some copies myself, but could not; and then, when any of our men wish to go abroad to practise, we can tell them what is probably expected of them, and they can fit themselves to pass the required examination, and the rest will be easy for them, I think.

